# Unravelling the genetic architecture of human complex traits through whole genome sequencing

**DOI:** 10.1038/s41467-023-39259-x

**Published:** 2023-06-14

**Authors:** Ozvan Bocher, Cristen J. Willer, Eleftheria Zeggini

**Affiliations:** 1 Institute of Translational Genomics, Helmholtz Zentrum München, German Research Center for Environmental Health, 85764 Neuherberg, Germany; 2 Department of Internal Medicine, Division of Cardiology, University of Michigan, Ann Arbor, MI 48109 USA; 3 Department of Human Genetics, University of Michigan, Ann Arbor, MI 48109 USA; 4 Department of Computational Medicine and Biostatistics, University of Michigan, Ann Arbor, MI 48109 USA; 5 Technical University of Munich (TUM) and Klinikum Rechts der Isar, TUM School of Medicine, Ismaninger Str. 22, 81675 Munich, Germany

**Keywords:** Next-generation sequencing, Genome-wide association studies, Rare variants

## Abstract

Whole genome sequencing has enabled new insights into the genetic architecture of complex traits, especially through access to low-frequency and rare variation. This *Comment* highlights the key contributions from this technology and discusses considerations for its use and future perspectives.

The field of human complex trait genetics has been enriched by high-throughput whole genome sequencing (WGS) technologies. WGS complements array-based genotyping by offering the opportunity to access most sequence in the genome and not only a set of known genetic variants. As sequencing costs gradually drop, an increasing number of study designs involve WGS approaches. Large sequencing projects have been undertaken in the general population that can be used as reference panels for genotype imputation in association studies, such as the Haplotype Reference Consortium (HRC^1^) project. Further initiatives, such as UKBioBank^2^ and TOPMed^3^, also make use of WGS technologies to sequence thousands of phenotypically-diverse individuals, providing resources of unprecedented scale to study the genetic architecture underlying complex diseases and traits^4^. Here, we comment on the progress and successes in the field heralded through WGS, as well as on future perspectives of this technology.

## Advantages of WGS

### Access to rare variation

The main advantage of using WGS approaches is direct access to genetic variation across the whole frequency spectrum, without first knowing where such variation occurs, as is required for array-based genotyping. Low frequency and rare variants are often not imputed accurately from reference panels (which are also frequently not a perfect population match), but can be detected by WGS. WGS offers more accurate and complete information capture of rare variation observed in sequenced individuals, their family members, and others with shared ancestry^5^. WGS-based studies have reported associations with rare variants of large effect size. These associations have been described in case-control studies, for example, in the TOPMed project where Zhao et al. identified a rare variant with a large effect on reduced lung function^6^, as well as in quantitative trait studies, for example, in the study by Benonisdottir et al., which identified nine rare variants associated with urinary biomarkers^7^. Detecting single-point rare variant associations requires very large sample sizes, especially if effect sizes are not large. To maximise the chance to detect rare variants associated with complex diseases and to consider genetic heterogeneity between individuals, rare variant association tests (RVAT) have been developed. These methods have enabled the detection of associations between medically relevant traits and an accumulation of rare variants in a chromosomal region, typically a single gene. For example, Gilly et al. described a cardioprotective rare variant burden in the *APOC3* gene, composed of exonic and splice variants, which could not be detected using imputation of genotype data^8^. In a larger study, by applying gene-based burden tests in over 17,000 binary phenotypes, Wang et al. identified over 1,700 significant associations, highlighting the importance of rare genetic variation in complex diseases^9^. Followed by functional investigation, these findings are bringing new insights into the biological mechanisms behind complex diseases. Nevertheless, biological interpretation can be more readily reached for the exome, on which the majority of RVAT have currently been applied.

Despite the description of several associations with rare variants, it is still unclear how much they contribute to the heritability of human complex traits. This proportion is especially hard to estimate for rare variants as they correspond to observations only in a few individuals resulting in high standard errors^10^. As common variants are present in more individuals, it is expected that they will contribute more to the phenotypic variance than rare variants. Apart from a few examples such as height^11^ or type 2 diabetes^12^, several studies have indeed shown that complex trait heritability due to rare variants is expected to be rather low. For example, by looking at 22 common traits, Weiner et al. showed that rare coding variants explain on average only 1.3% of the overall phenotypic variance, ranging from 0.4% for asthma to 3.6% for height^13^. While rare variants will unlikely explain all the remaining phenotypic variability of complex traits, they can also be useful for prediction^14^. For instance, a study to predict haemoglobin A1C levels showed that the integration of many rare variants into prediction scores could lead to the identification of a substantial number of undiagnosed type 2 diabetes cases^15^.

### Ancestry-diverse studies

A further important benefit of WGS is the investigation of under-represented populations that have not been well characterised by currently available sequencing data, in which rare or population-enriched variation is therefore not accurately described^16^. For example, using genotyping and low-depth sequencing in 6,400 individuals from the Uganda population, numerous associations were identified with complex traits, including both novel findings and associations at previously reported loci but with different allelic effects^17^. Similarly, sequencing followed by imputation of the Icelandic population has resulted in novel insights, including an association between a splice variant in *RPL3L* and atrial fibrillation^18^. Considering sub-populations within Europeans is also of interest: in Norwegians, low-depth sequencing followed by a custom genotyping array performed on 70,000 individuals resulted in new associations, e.g., between *ZNF529* p.K405X and LDL-C^19^. A GWAS performed in the Finnish population on 1,932 phenotypes found 2,491 significant associations, including newly associated variants that could be identified due to their higher frequency in the Finnish population. For example, an intronic variant of *TNRC18* strongly associated with IBD but almost absent from other European populations^20^. WGS in diverse populations represents one of the most active areas of research, as getting an overview of the genetic architecture in diverse populations will enable better comprehension of complex diseases as well as the differences in effect direction and sizes of the associated variants that are observed across populations^10^.

## Considerations in WGS-based studies

### Challenges in study design

Genotype-based GWAS is an established field where power has been shown to clearly depend on the sample size and the detectable genetic effect^21^, however, planning the design of a sequencing-based study can be more challenging. Li et al. showed that power indeed also depends on read depth and distribution^22^. In addition, the power of RVAT is less straightforward to estimate compared to single-point association analysis, because it depends on additional parameters such as the filtering strategy used to select qualifying variants and their directions of effect. Power is a major driver of the success of a study, and multiple software packages are available to estimate the expected power of WGS-based studies, as reviewed in Li et al.^22^. Conducting studies in diverse populations can provide useful insights into the genetics of complex diseases. Furthermore, power to detect associations can be boosted by studying isolated populations, in which variant frequencies and effect sizes may be larger^8,18,20^. As the majority of WGS projects to date have been focused on European populations, conducting sequencing-based studies in under-represented populations is expected to be of benefit^10^ and represents an important direction for future WGS applications.

### Determination of WGS approach

WGS-based studies can take various forms, for which the optimal choice will depend on several parameters including the population under study, the biological hypothesis investigated and the computational and financial resources available, with the cost of WGS being its largest disadvantage. These forms include WGS coupled to imputation in genome-wide association studies, cohort-wide low- or very low-depth WGS, and deep WGS (Box 1). When the interest is in related samples or under-represented populations, low and very-low depth WGS approaches may be relatively more efficient compared to when examining well-studied populations. For example, Tran et al. performed low-depth sequencing to genetically describe the Vietnamese population and reported five disease-associated pathogenic variants with higher allelic frequencies than in other populations^23^. Low-depth sequencing has indeed been shown to be more efficient than classical imputation-coupled array designs in detecting GWAS signals, primarily due to a more complete assessment of genomic variation, especially in ancestries with poor coverage in existing imputation panels^24^. This was illustrated in a study from Gilly et al. in an isolated Greek population where twice as many variants were detected using very low-depth WGS as compared to classical imputed array genotyping data, and a vast majority of which were rare variants, leading to a twofold increase in the number of association signals^25^. Nevertheless, low-depth WGS shows decreased accuracy when studying rare variation (frequency lower than 1%) in the genome compared to low-frequency (frequency between 1 and 5%) and common variants. To identify such variation, medium-depth designs can be applied, or high-depth sequencing for detecting indels and ultra-rare variation, such as singletons, with high accuracy^26^. While high-depth WGS has proven to be useful, for example, in the study by Wessel et al., which described the contribution of rare non-coding variants to type 2 diabetes^12^, it remains expensive, especially for large cohorts. A solution to perform high-depth sequencing at a lower cost would be to focus on coding parts of the genome by using whole exome sequencing (WES). The lower cost associated with WES would enable the inclusion of more individuals in the study and therefore an increase in the power to detect genetic variants that reside in genes and are associated with human complex traits, as illustrated in the study by Wang et al.^9^. Nevertheless, using WES instead of WGS misses genetic variation in the non-coding genome (or any gene with poor coverage in whole exome studies), which has been shown to play an important role in complex diseases^27^. Finally, emerging technologies such as long-read sequencing offer the possibility to access genome-wide structural variants which have been found to have an impact on complex phenotypes as highlighted by Beyter et al. on LDL cholesterol levels and height^28^. This approach provides additional advantages, such as easier assembly and mapping of genomes, but remains the most expensive sequencing technology, preventing its use in large cohorts.

Box 1 Overview of the different sequencing techniques currently available White boxes correspond to coding exons and thin black lines to sequencing reads. The sequencing depth is represented at the bottom of each graphic by brown shades. Pros and cons of the different depths and genome coverage are highlighted.
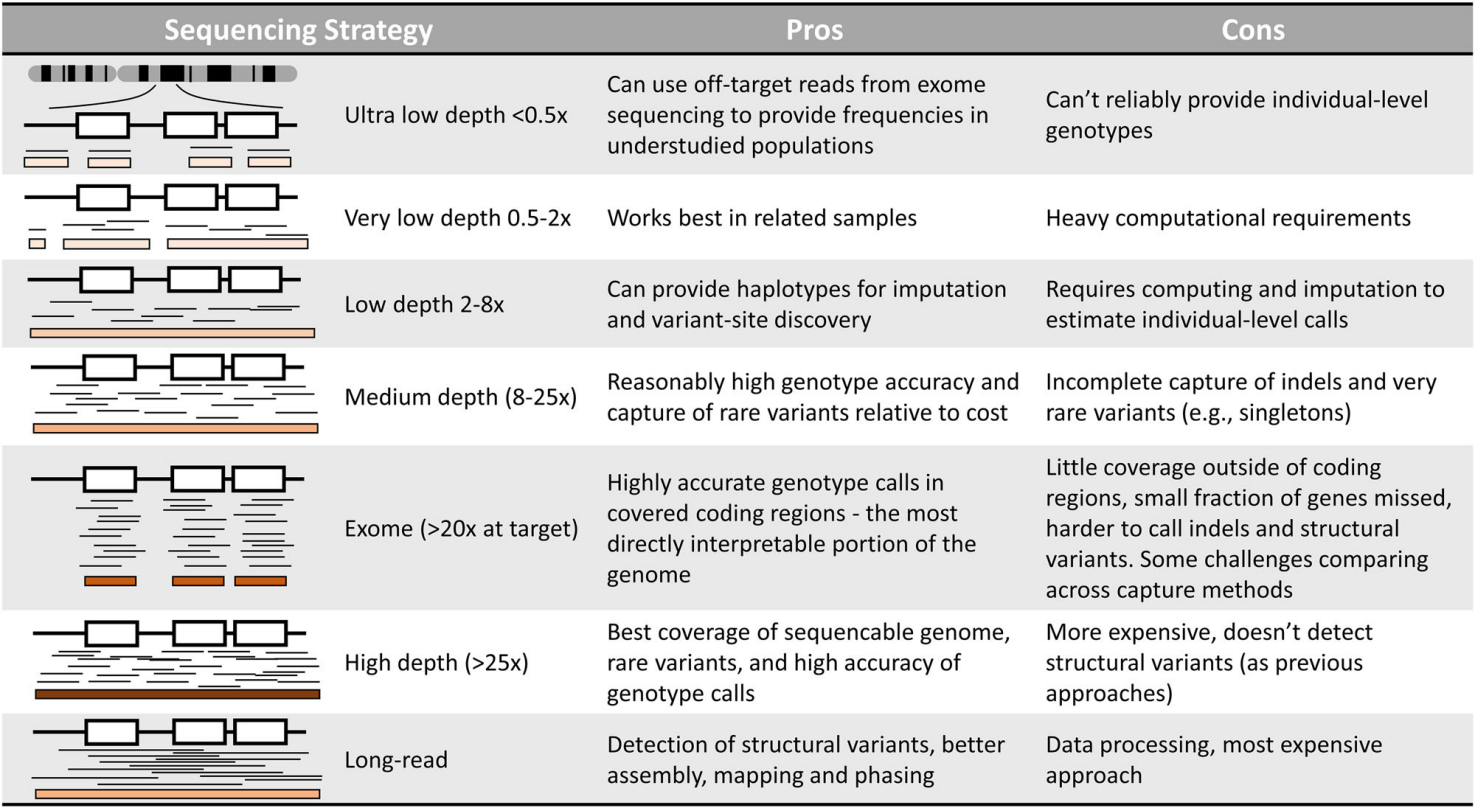

### Disadvantages compared to genotype-based studies

Despite recent progress, the cost of WGS remains prohibitive for most large-scale studies. Genotyping coupled to imputation can retrieve most common variation in the genome^29^. The use of cost-efficient array-based technologies enables increasing sample sizes, which in turns results in the identification of further associations with common and low-frequency variants. One striking example is the study by Yengo et al. on over 5 million individuals, which has described all of the genetic heritability of height due to common variants^30^. The contribution of common variants to the genetic architecture of human complex traits is still not fully understood and array-based technologies will continue to be useful in filling this gap. In addition, while sequencing will continue to contribute to obtaining the whole picture of the genetic architecture of complex traits, it is likely that translation into the clinic to screen for polygenic risk will focus on array-genotyping approaches rather than on sequencing at first.

## Perspectives and conclusion

WGS has made an important contribution to the understanding of genetics underlying complex traits, especially in under-represented populations, and through rare variation. Functional interpretation of association signals arising from WGS remains more challenging in the non-coding genome compared to the exome. Even if single-point associations have been described with rare and common variants in these regions, it is still arduous to biologically characterise these association signals. Combining association results from WGS with functional information at multiple levels, using, for example, other omics data such as transcriptomics, open chromatin, methylation, metabolomics or proteomics, has been shown to help in the interpretation of the associated signals^31^. Similarly, using RVAT in non-coding regions of the genome is not straightforward, despite affording higher power to detect genetic associations with rare variants^32^. Novel statistical methods are therefore needed which, for example, consider functional information across the non-coding genome^33,34^. WGS studies will remain useful in the future as a tool to explore the genetic underpinning of complex diseases, especially in combination with emerging functional data and their integration at multiple levels. As the cost of WGS is dropping and given the exciting prospect of long-read WGS at scale, these technologies will become increasingly accessible and will enable the description of genetic variation in hitherto understudied populations. As our understanding of the non-coding genome continues to improve, and with the further development of powerful methods to integrate functional information in rare variant association testing approaches, WGS will hopefully lead to a better and more accurate comprehension of complex diseases. In the future, it is anticipated that WGS-informed clinical decisions and interventions will accelerate personalised medicine in the wider field of complex diseases, following recent successes in cancer and rare disease, such as monogenic forms of cardiomyopathy^35,36^. To achieve these goals in a globally equitable fashion, WGS of diverse populations should remain a high priority going forward.
